# Supplementary material for: Sorption to soil, biochar and compost: is prediction to multicomponent mixtures possible based on single sorbent measurements?
Source: PeerJ. 2018 Jun 11;6:e4996. doi: 10.7717/peerj.4996 (PMC6001708; doi:10.7717/peerj.4996)
Supplement: Supplemental Information 1 [file peerj-06-4996-s001.pdf]

Sorption to soil, biochar and compost: is prediction to multicomponent mixtures possible based on single sorbent measurements?

Melanie Kah[a,b*], Gabriel Sigmund[a,c], Pedro Luis Manga Chavez[a], Lucie Bielská[d], Thilo Hofmann[a*]

[a] University of Vienna, Department of Environmental Geosciences and Environmental Science Research Network, Althanstrasse 14, 5 UZA2, 1090 Vienna, Austria

[b] Commonwealth Scientific and Industrial Research Organisation (CSIRO), Waite Campus, Locked Bag No 2, Glen Osmond, SA 5064, Australia

[c] Present address: Chair of Analytical Chemistry and Water Chemistry, Technical University of Munich, Marchioninistrasse 17, 81377 Munich, Germany

[d] RECETOX, Faculty of Science, Masaryk University, Kamenice 5, 625 00 Brno, Czech Republic

Melanie.kah@univie.ac.at, thilo.hofmann@univie.ac.at

Contents

Table S1. Main properties of the two soils studied S2

Table S2. Main properties of the four biochars studied S2

Table S3. Calculated organic carbon content (OC%) of the mixtures. S2

Table S4. Freundlich isotherm parameters for the series of four biochars. S3

Table S5. Freundlich isotherm parameters for the series of sorbents and their mixtures........ S3

Figure S1. Scanning electron microscope images of the biochars produced from miscanthus straw S4

Figure S2. Scanning electron microscope images of the biochars produced from soft wood S4

Figure S3. Sorption isotherm of pyrene to compost measured by batch and the POM methods S5

Figure S4. Sorption isotherms of pyrene to the biochar, compost and their mixture (log scale) S5

Figure S5. Sorption isotherms of pyrene to the clay loam and sandy loam before and after addition of 5% biochar, 10% compost or both (log scale) S6

Prepared in May 2018.

Prepared

Table S1. Main properties of the two soils studied

	Sand (%)	Silt (%)	Clay (%)	OC (%)	pH (0.01M $CaCl_2$)
**Clay Loam**[a]	45	28	27	1.7	6.2
**Sandy Loam**	64	21	15	1.0	4.2

[a] Following the USDA textural classes

Table S2. Main properties of the four biochars studied

						N_2 (NLDFT[b])		CO_2 (GCMC[c])	
	C (%)	H/C	O/C	Ash (%)	pH [a]	Surface Area (m^2/g)	Pore volume (cc/g)	Surface Area (m^2/g)	Pore volume (cc/g)
**MSP550**	75.41	0.38	0.09	12.15	9.77	16	0.024	320	0.092
**MSP700**	79.18	0.19	0.07	11.55	9.72	38	0.029	369	0.103
**SWP550**	85.52	0.39	0.09	1.25	7.91	31	0.029	375	0.118
**SWP700**	90.21	0.24	0.05	1.89	8.44	173	0.119	449	0.132

[a] measured in 0.01M $CaCl_2$
[b] Nonlocal Density Functional Theory
[c] Grand Canonical Monte Carlo analysis

Table S3. Calculated organic carbon content (OC%) of the mixtures.

	Clay Loam + B	Clay Loam + C	Clay Loam + B + C	B + C	Sandy Loam + B	Sandy Loam + C	Sandy Loam + B + C
**OC%**	5.4	2.7	6.4	32.6	4.7	2.0	5.7

Table S4. Freundlich isotherm parameters K_f (sorption affinity) and n (linearity parameter) for pyrene sorption measured by batch to the series of four biochars derived from miscanthus (MS) and soft wood (SW) at 550 and 700 °C.

s.e. is the standard error associated with nonlinear fit, and N is the number of data point considered.

	K_f (μg/kg)/(μg/L)^n	*s.e.*	n	*Std.*	r^2	N
**MS550**	231472	*11530*	0.67	*0.05*	0.95	18
**MS700**	180889	*9677*	0.55	*0.05*	0.92	18
**SW550**	47216	*2789*	0.48	*0.03*	0.97	18
**SW700**	74397	*3618*	0.45	*0.03*	0.96	18

Table S5. Freundlich isotherm parameters K_f (sorption affinity, (μg/kg)/(μg/L)^n) and n (linearity parameter) for pyrene sorption to the series of sorbents and their mixtures measured by a combination of batch and passive sampling methods (biochar was only measured by batch). K_d and K_{OC} values were calculated at 0.02 and 2 μg/L to allow direct comparisons.

s.e. is the standard error associated with nonlinear fit, and N is the number of data point considered. CL and SL refer to Clay Loam and Sandy Loam, respectively.

	K_f	*s.e.*	n	*s.e.*	r^2	N	K_d0.02	K_d2	K_{OC}0.02	K_{OC}2
Biochar (B)[a]	231472	*11530*	0.67	*0.05*	0.95	18	8.32E+05	1.85E+05	1.11E+06	2.46E+05
Compost (C)	30780	*410*	0.89	*0.01*	1.00	29	4.75E+04	2.85E+04	7.57E+04	7.80E+03
B + C	44513	*770*	0.73	*0.02*	0.99	27	1.26E+05	3.70E+04	3.87E+05	1.14E+05
Clay Loam	2322	*74*	0.75	*0.02*	1.00	25	6.18E+03	1.95E+03	3.55E+05	1.12E+05
CL + B	6482	*74*	0.69	*0.01*	0.99	29	2.19E+04	5.23E+03	4.05E+05	9.67E+04
CL + C	5050	*79*	0.90	*0.02*	0.99	28	7.41E+03	4.72E+03	2.74E+05	1.74E+05
CL + B + C	9978	*213*	0.89	*0.03*	0.98	31	1.53E+04	9.25E+03	2.41E+05	1.45E+05
Sandy Loam	1699	*107*	0.73	*0.03*	0.99	24	4.87E+03	1.41E+03	4.87E+05	1.41E+05
SL + B	4168	*110*	0.69	*0.03*	0.98	29	1.38E+04	3.37E+03	2.93E+05	7.18E+04
SL + C	4926	*89*	0.88	*0.03*	0.99	29	7.84E+03	4.54E+03	3.84E+05	2.22E+05
SL + B + C	6990	*169*	0.81	*0.03*	0.97	30	1.46E+04	6.13E+03	2.55E+05	1.07E+05

[a] Sorption to the biochar (MS550) was only measured by batch.

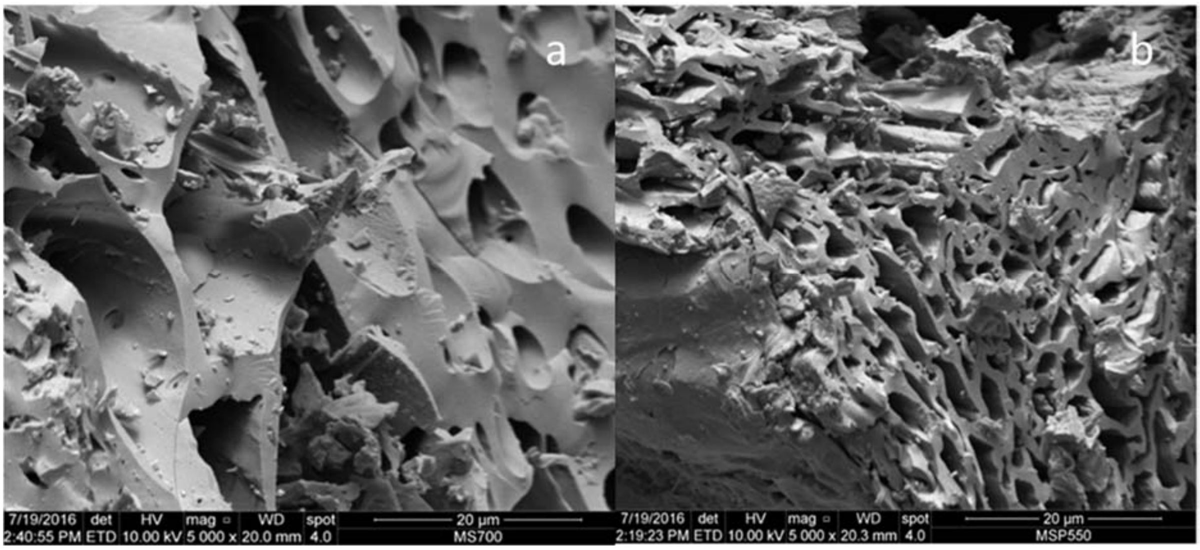

Figure S1. Scanning electron microscope images of the biochars produced from miscanthus straw at (a) 700°C and (b) 550°C

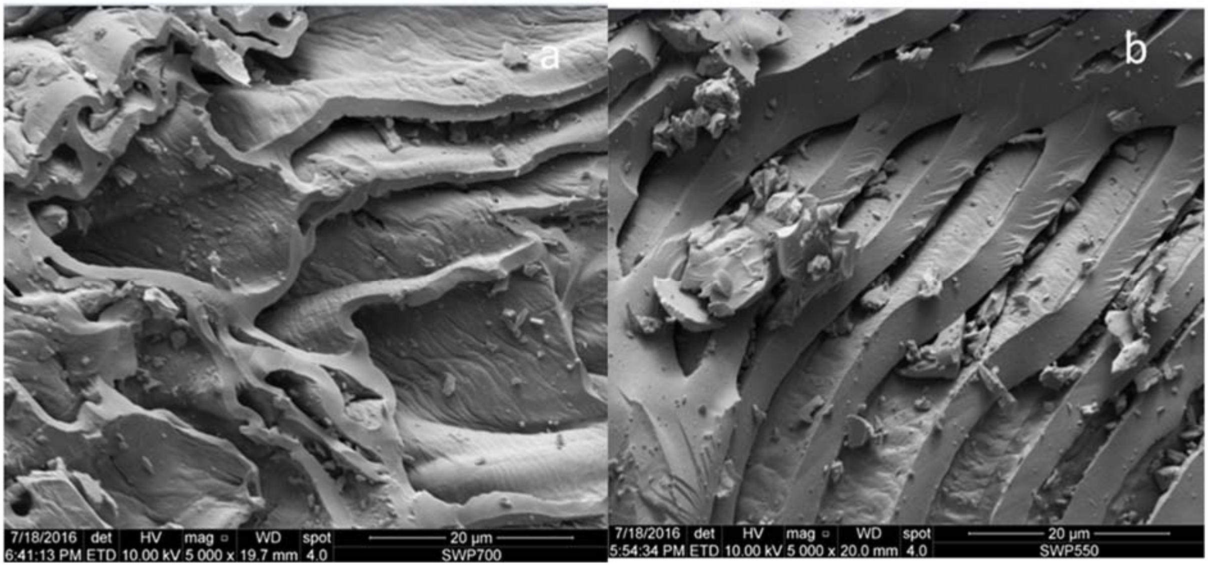

Figure S2. Scanning electron microscope images of the biochars produced from soft wood at (a) 700°C and (b) 550°C

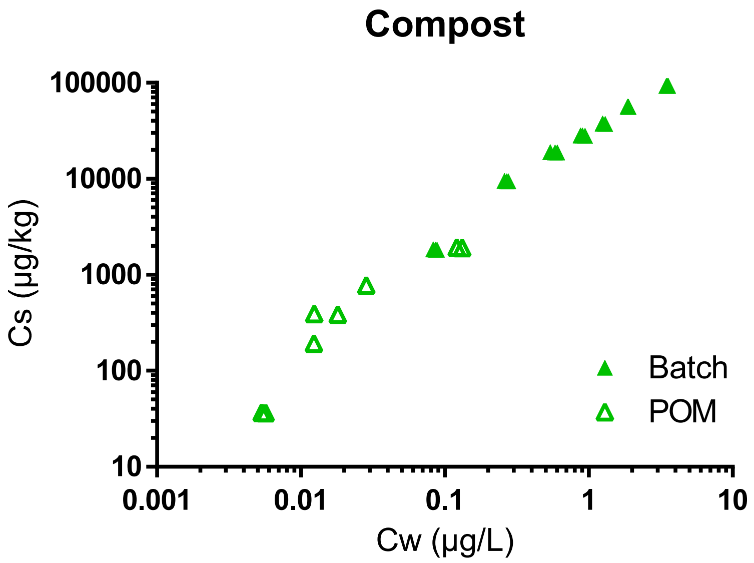

Figure S3. Sorption isotherm of pyrene to compost measured by batch (full symbols) and the POM method (open symbols).

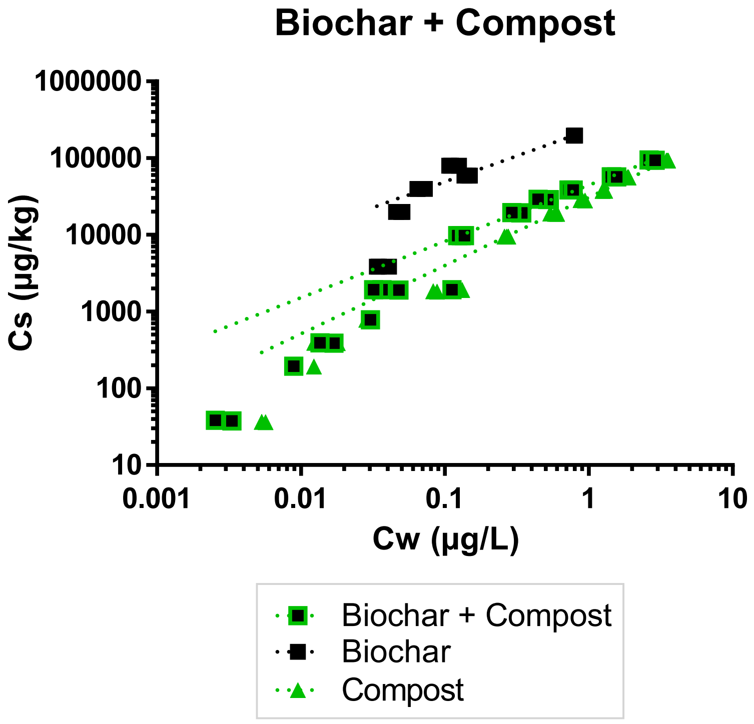

Figure S4. Sorption isotherms of pyrene to the biochar (black squares), compost (green triangles), and their mixture (1:2, green and black squares) represented in a logarithmic scale.

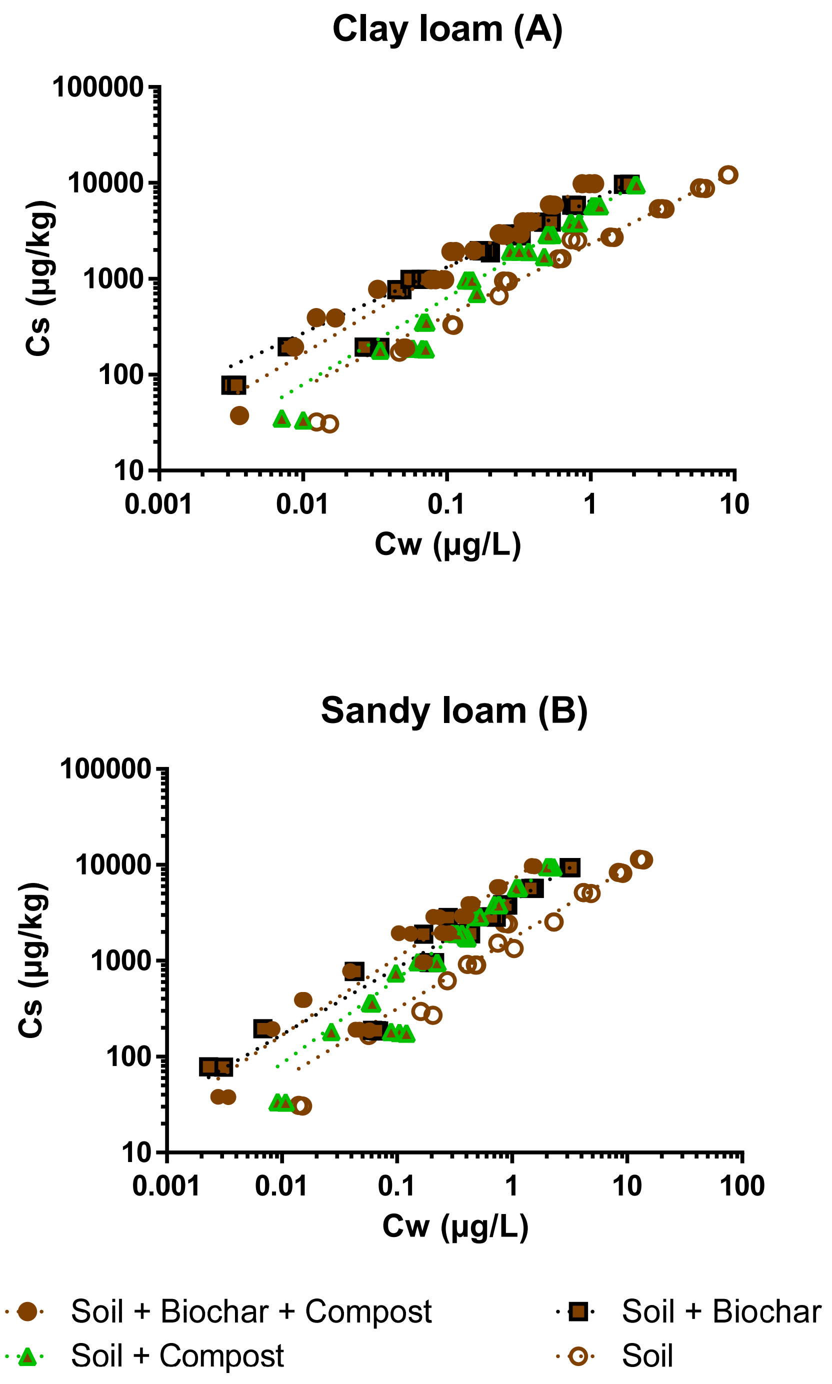

Figure S5. Sorption isotherms of pyrene to the clay loam (A) and sandy loam (B) before (open brown circles) and after addition of 5% biochar (black squares), 10% compost (green triangles) or both (full brown circles).
